# *Bacteroides fragilis* Supplementation Deteriorated Metabolic Dysfunction, Inflammation, and Aorta Atherosclerosis by Inducing Gut Microbiota Dysbiosis in Animal Model

**DOI:** 10.3390/nu14112199

**Published:** 2022-05-25

**Authors:** Guoxiang Shi, Yubi Lin, Yuanyuan Wu, Jing Zhou, Lixiang Cao, Jiyan Chen, Yong Li, Ning Tan, Shilong Zhong

**Affiliations:** 1 Guangdong Provincial Key Laboratory of Coronary, Department of Pharmacy, Heart Disease Prevention, Guangdong Cardiovascular Institute, Guangdong Provincial People’s Hospital, Guangdong Academy of Medical Sciences, School of Medicine, South China University of Technology, Guangzhou 510080, China; shiguoxiang168@163.com (G.S.); linyb23@mail.sysu.edu.cn (Y.L.); wyy0318@foxmail.com (Y.W.); zhoujing@gdph.org.cn (J.Z.); chen-jiyan@163.com (J.C.); 2 Department of Cardiology, The First Affiliated Hospital of Nanchang University, Jiangxi Hypertension Research Institute, Nanchang 335100, China; 3 The First Dongguan Affiliated Hospital, Guangdong Medical University, Dongguan 523710, China; 4 School of Medicine, Sun Yat-sen University, Guangzhou 510317, China; lssclx@163.com; 5 Department of Surgery, Guangdong Provincial People’s Hospital, Guangdong Academy of Medical Sciences, Guangzhou 510317, China

**Keywords:** gut microbiota, dysbiosis, atherosclerotic, metabolic syndrome

## Abstract

Background: The gut microbial ecosystem is an important factor that regulates host health and the onset of chronic diseases, such as inflammatory bowel diseases, obesity, hyperlipidemia, and diabetes mellitus, which are important risk factors for atherosclerosis. However, the links among diet, microbiota composition, and atherosclerotic progression are unclear. Methods and results: Four-week-old mice (^-/-^ mice, C57Bl/6) were randomly divided into two groups, namely, supplementation with culture medium (control, CTR) and *Bacteroides fragilis* (BFS), and were fed a high-fat diet. The gut microbiota abundance in feces was evaluated using the 16S rDNA cloning library construction, sequencing, and bioinformatics analysis. The atherosclerotic lesion was estimated using Oil Red O staining. Levels of CD36, a scavenger receptor implicated in atherosclerosis, and F4/80, a macrophage marker in small intestine, were quantified by quantitative real-time PCR. Compared with the CTR group, the BFS group showed increased food intake, fasting blood glucose level, body weight, low-density lipoprotein level, and aortic atherosclerotic lesions. BFS dramatically reduced *Lactobacillaceae* (LAC) abundance and increased *Desulfovibrionaceae* (DSV) abundance. The mRNA expression levels of CD36 and F4/80 in small intestine and aorta tissue in the BFS group were significantly higher than those in the CTR group. Conclusions: gut microbiota dysbiosis was induced by BFS. It was characterized by reduced LAC and increased DSV abundance and led to the deterioration of glucose/lipid metabolic dysfunction and inflammatory response, which likely promoted aorta plaque formation and the progression of atherosclerosis.

## 1. Introduction

The gut microflora microenvironment is essential in maintaining the normal physiological function of humans. The destructive composition and delicate balance of gut microflora are associated with many diseases, such as Crohn’s disease and metabolic syndrome [1,2,3,4]. Gut microflora dysbacteriosis also aggravates the pathogenic process of obesity, hypertension, lipid metabolic disorder, diabetes mellitus, and insulin resistance, which are important risk factors of atherosclerosis [5,6,7,8]. The harmful gut microflora, such as *Chlamydia pneumoniae*, *Helicobacter pylori*, and *Porphyromonas gingivalis*, infect macrophages to promote foam cell formation, leukocyte recruitment, smooth muscle proliferation, and lesion progression, thereby ultimately facilitating the aggravation of atherosclerosis [9]. According to the study of Emoto, the alterations of gut microbiota were linked to the incidence of coronary artery disease (CHD) [10]. Interestingly, in our previous study, we demonstrated that the gut microflora species and their abundance in patients with CHD are remarkably different from those in patients without CHD. Patients with CHD exhibit increased *Ruminococcus* and *bactericides* abundance and decreased *Prevotella* abundance. For the first time, this study found that *Bacteroides fragilis* (*B. fragilis*) abundance is positively associated with hip circumference and dramatically increased in patients with CHD [11]. Some studies reported that *B. fragilis* supplementation (BFS) increases the risk of diabetes mellitus [12,13], suggesting the potentially important role of *B. fragilis* in atherosclerosis. However, the pathogenesis of gut microbiota and *B. fragilis*’ contribution to atherosclerosis remain unclear. In the present study, we aimed to explore whether and how BFS affects the diet, metabolic functions, intestinal environment, inflammation, and atherosclerotic lesion in the aorta of mice.

## 2. Materials and Methods

### 2.1. Animal Group and Intervention

Twenty *Aope^-/-^* mice (C57Bl/6, 4-weeks-old, male, and SPF-grade) were purchased from the Experimental Animal Center of Peking University and fed in accordance with the guidelines for the rearing and use of laboratory animals issued by the National Institutes of Health (NIH publication NO. 23–85, revised 1996) and approved by the Hospital Animal Care Committee.

An SPF-grade feeding environment was used. Five animals were kept in a cage and given free access to water and high-fat diet (DIO series diets, 60% H10060; calorie, 5.24 kcal/g) [14]. The whole experiment lasted for 12 weeks. Mice were housed in a 12 h light/dark cycle. Twenty mice were randomly divided into two groups (*n* = 10 mice), as follows: (1) The control group was fed with the medium (used for *B. fragilis* propagation); and (2) the experimental group was fed with *B. fragilis* ATCC 25285 by gavage (*B. fragilis* supplementation, BFS) once daily for three consecutive days at 0.3–0.4 mL each time. The colony concentration was 10^9^–10^10^/mL. Fresh feces were collected 1 day before BFS and every 2 weeks after supplementation.

### 2.2. Strain Recovery and Isolation Culture

Anaerobic bacteria and anaerobic blood culture medium were mixed quickly and the suspension was pumped into the corresponding culture medium and cultured on the shaking table for 24–48 h. When bacteria grew turbid in the culture medium, the *B. fragilis* culture medium was aseptically pumped and placed in the BBE culture medium. After partition lineation, anaerobic culture was conducted immediately in a Genbag anaerobic gas production bag. All bacteria were cultured in a 5% CO_2_ incubator at 36 °C for 24–48 h.

### 2.3. Specimen Collection and Processing

During the experiment, the body weight, diet, and drinking water consumption of mice were measured weekly. The fasting blood glucose after fasting for 8 h was detected before the intervention and every 2 weeks after the intervention. Blood samples were collected from the tail vein of the mice, and blood glucose was measured using a portable blood glucose meter and blood glucose test paper. During the feeding period, fresh feces of mice were collected every week and stored in a refrigerator at −80 °C.

Mice underwent cardiac color Doppler ultrasound examination one day before disposal. At the end of the experiment, animals were sacrificed after fasting overnight. Venous blood was obtained from the retro-orbital venous plexus. The thoracic cavity was opened, and PBS solution was injected into the left ventricle until the heart and blood vessel became white.

### 2.4. Biochemical Assays in Plasma

The lipid profiles, including total cholesterol, high-density lipoprotein-cholesterol (HDL-C), low-density lipoprotein-cholesterol (LDL-C), and triglyceride concentrations, were measured in the laboratory of Guangdong General Hospital (7170s automatic biochemical analyzer, Hitachi, Tokyo, Japan).

### 2.5. Aortic Staining with Oil Red

Aortas were isolated from animal models under the anatomical microscope, and the atherosclerotic lesion size was quantified using computerized image analysis after staining the entire aorta with Oil Red O. Atherosclerosis severity was determined by the percentage of lipid plaque infiltration and total blood vessel area.

### 2.6. Sampling DNA Extraction, 16S rDNA Cloning Library Construction and Sequencing

Fresh fecal samples (before supplementation and at the 2nd week after supplementation) and cecum content (12th week after supplementation) were collected from mice and quickly placed in an anaerobic culture tank. The ZR Fecal DNA MiniPrep™ (Product Code: Catalog No. D6010) test kit was used to extract DNA from feces. The total DNA of each fecal sample from mice was amplified with primers (Table 1), which corresponded to the conserved regions of the 5′ and 3′ ends of the 16S rDNA gene. The DNA extracted from feces was amplified by PCR with primers from the Promega Company in strict accordance with the instructions. Finally, clones were randomly selected for sequencing, and a clone library was constructed. Positive clones were selected and sent to the Huada Gene Center (BGI, Shenzhen, China) for sequencing. The clones in each library were sequenced for full-length 16S rDNA by the ABI 3730xl Sequencer (Applied Biosystems, Waltham, MA, USA) with vector primers SP6/T7.

### 2.7. Gut Microbiota Bioinformatics Analysis

The BLASTN software (Annapolis, MD, USA) was used to compare the unique tag sequence with the 16S rDNA in the database and to annotate the tag species. The number of OTUs with 97% similarity was calculated using the mothur software (Lansing, MI, USA). OTU species were annotated on the basis of the mode principle, and alpha diversity indices, including ACE value, Chao1 value, and Simpson and Shannon indices, were determined. The mothur (v.1.11.0) software (Lansing, MI, USA). drew the relative proportion of the OTUs of 16S rDNA sequence and the rarefaction curve to evaluate whether the sequencing amount was enough to cover all groups, thereby indirectly reflecting the richness of species in the sample. By comparing the abundance differences of the same species in different samples, species with significant differences in different samples were selected. The beta diversity of different samples at the best classification level was calculated. In accordance with the relative content of each species at the best classification level, the principal component analysis (PCA) was used to identify the species that contributed to the differences among samples. The R (v.2.9.1) software (Auckland, New Zealand) was used to calculate the distance between samples and cluster analysis was used to assess the similarity of samples’ species composition at the family level.

### 2.8. Inflammatory Factors Detected by qRT-PCR

Total RNA was extracted using Trizol reagent added with RQ1 RNase-Free DNase (Invitrogen Life Technologies, Carlsbad, CA, USA). The RNA was reverse-transcribed to cDNA by using the PrimeScript RT Reagent kit (Takara, Kyoto, Japan) in accordance with the manufacturer’s protocol. The cDNA was then quantified by qPCR on the ViiA7 instrument (Life Technologies, NewYork, NY, USA) with appropriate primers (Table 2) by using the SYBR Premix Ex TaqII (Takara, Kyoto, Japan). The mRNA level of the GAPDH housekeeping gene served as a control.

### 2.9. Statistical Analysis

Data were expressed as mean ± standard deviation ($\bar{x}$ ± SD) and analyzed using the SPSS17.0 statistical software. The two groups were compared using the independent sample *t*-test. *p* < 0.05 was defined as statistically significant.

## 3. Results

### 3.1. Diet and Drinking Water

**Figure 1:**
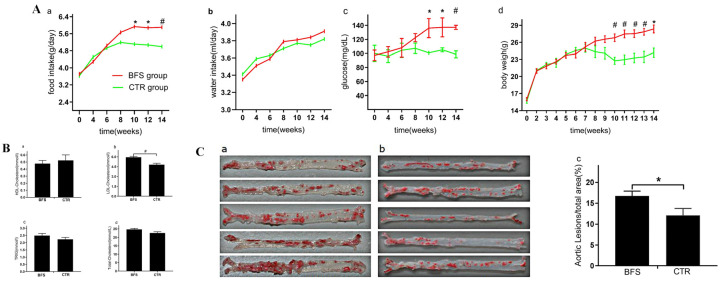
The differences of food intake, glucose, body weight, and lipid level between *B. fragilis* and control groups. (**A**) (a–d): Food intake, water intake, fasting blood glucose, and body weight varied with age. (**B**) (a–d): Effect of *Bacteroides fragilis* supplementation (BFS) on blood lipid in ^-/-^ mice. (**C**) (a–c): Gross Oil Red O staining and atherosclerotic lesion area of aorta surface in each group. (**C**) (a): The atherosclerosis lesion in BFS group. (**C**) (b): The atherosclerosis lesion in CTR group (control group). * *p* < 0.05, ^#^ *p <* 0.01 compared with the CTR group.

The food intake of mice increased with age and tended to be stable 10 weeks after supplementation (Figure 1A(a)). No significant difference was found in the food intake between the two groups before the first 6 weeks, but the food intake of the BFS group increased faster than that of the control group at 6 weeks and especially at 10 weeks after supplementation. The average food intake of each mouse per day in the BFS group gradually increased compared with that in the control group, starting from the 10th week. As shown in Figure 1A(b), the amount of drinking water consumed by the two groups increased with age and stabilized at 10 weeks after supplementation. No statistical difference was observed between the two groups during the experiment.

### 3.2. Fasting Blood Glucose

Figure 1A(c) showed that the fasting blood glucose of the BFS group increased with age and stabilized at the 8th week after BFS. The fasting blood glucose of the control group remained stable during the experiment. No significant difference was observed in the fasting blood glucose between the two groups in the first 8 weeks after BFS. Starting from the 10th week, the fasting blood glucose of the BFS group was significantly higher than that of the control group (7.58 ± 1.78 vs. 5.12 ± 0.33, *p* < 0.05).

### 3.3. Body/Heart Weight and Cardiac Systolic Function

Figure 1A(d) shows that the body weights increased with age significantly in the first 8 weeks and stabilized at the 8th week. No significant difference was observed between the two groups in terms of body weight in the first 7 weeks after supplementation. At 7 weeks, the body weight of the BFS group increased compared with that of the control group. Starting from the 10th week, the body weight of the BFS group significantly increased compared with that of the control group (26.82 ± 1.50 vs. 22.76 ± 1.38 g, *p <* 0.01). At the end of the experiment, all mice survived without withdrawal. Compared with the control group, the body and heart of the BFS group were heavier at the 12th week (Table 3), but no significant difference was observed in the ratio of heart/body weight between the two groups. Additionally, no significant difference was observed in the cardiac systolic function between the two groups.

### 3.4. Blood Lipid Level

Compared with the control group (Figure 1B(a)), the BFS group did not show any difference in HDL-C level (0.52 ± 0.14 vs. 0.48 ± 0.10 mmol/L, *p* > 0.05). The levels of LDL-C (5.94 ± 0.31 vs. 4.81 ± 0.37, *p* < 0.01) in the BFS group were higher than those in the control group (Figure 1B(b)). Compared with the control group (Figure 1B(c)), the BFS group did not show any difference in triglyceride level (TRIG: 2.49 ± 0.33 vs. 2.23 ± 0.24 mmol/L, *p* > 0.05). The levels of total cholesterol (TC: 24.71 ± 1.40 vs. 22.58 ± 1.27 mmol/L, *p* > 0.05) in the BFS group illustrated increasing trends compared with those in the control group.

### 3.5. Aorta Atherosclerotic Lesion

The aortic surface was stained using the Oil Red O method to observe the extent of atherosclerotic lesions in both groups (Figure 1C(a,b)). Compared with the control group (Figure 1C(c)), significantly increased extent of atherosclerotic lesions on the aortic surface was observed in the BFS group (12.08% ± 3.72% vs. 16.75% ± 3.69%, *p* < 0.05).

### 3.6. Inflammatory Marker Expression

**Figure 2:**
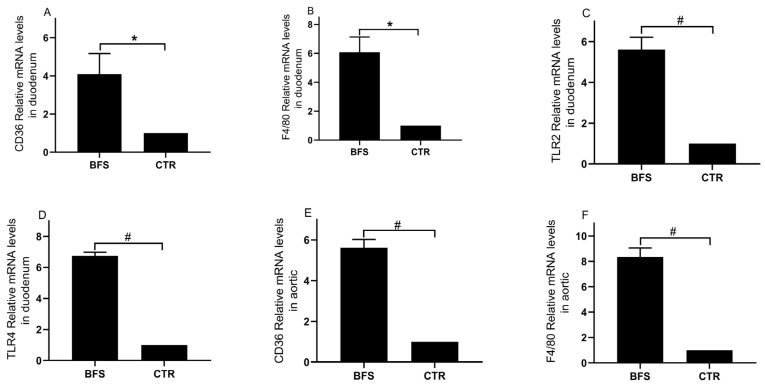
The mRNA expressions of CD36, F4/80, TLR2, and TLR4 in the duodenum and aortic tissue of mice; (**A**–**D**), CD36, F4/80, TLR2, and TLR4 mRNA expression levels in duodenum tissue, respectively; (**E**,**F**), CD36 and F4/80 mRNA expression levels in aortic tissue, respectively. * *p* < 0.05, ^#^ *p* < 0.01 compared with the CTR group.

The mRNA expression levels of TLR2, TLR4, CD36, and F4/80 in the duodenum and aorta tissues were analyzed using qRT-PCR. Compared with the control group, the BFS group showed significantly increased mRNA expression levels of CD36 (Figure 2A, 1.00 ± 0.00 vs. 5.10 ± 2.50, *p* < 0.05), F4/80 (Figure 2B, 1.00 ± 0.00 vs. 6.07 ± 3.34, *p* < 0.05), TLR2 (Figure 2C, 1.00 ± 0.00 vs. 5.61 ± 0.60, *p* < 0.01), and TLR4 (Figure 2D, 1.00 ± 0.00 vs. 6.74 ± 0.24, *p* < 0.01) in the duodenum tissue. The mRNA expression levels of CD36 (Figure 2E, 1.00 ± 0.00 vs. 5.61 ± 0.41, *p* < 0.01) and F4/80 (Figure 2F, 1.00 ± 0.00 vs. 8.34 ± 0.71, *p* < 0.01) in the BFS group’s aorta tissue were also significantly elevated.

### 3.7. Total Bacterial Abundance and Diversity

**Figure 3:**
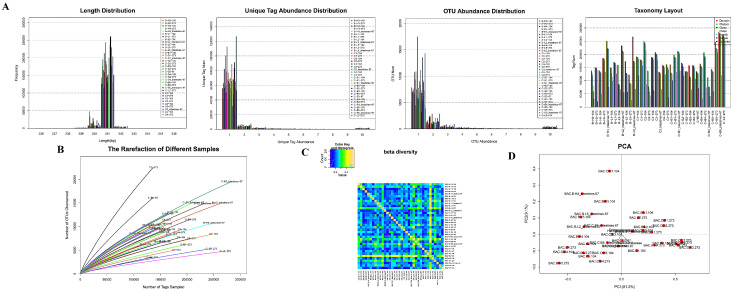
The tags abundance statistics: (**A**): The length distribution of tags in each group; The tags abundance distribution in each group; The OUT distribution in each group; The number of tags in each classification level of each sample was statistically analyzed. (**B**): The dilution curve of each group. (**C**): The beta diversity of samples in each group. (**D**): The PCA diagram of samples in each group.

The abundance of most unique tags was 1 (Figure 3A,B). The OTU abundance statistics showed that the number of tags in the OTU from most samples was solely 1 (Figure 3C). The OTU species annotation (Figure 3D) indicated that more than 41% of tag sequences in samples could be annotated to the family level, whereas the number of tag sequences that could be annotated to the genus level was less than 14%. Therefore, the family level was chosen as the best classification level for the samples. At this level, a balance was reached for the two factors of species grade and the number of annotated tags.

The alpha diversity analysis showed no significant difference in the species of each group. The dilution curves of samples reached a plateau period or tended to be flat (Figure 3E), indicating equal levels of diversity within each sample. The beta diversity value of samples in the two groups was relatively large (Figure 3F), indicating that a significant difference in microbial signatures existed among the samples. PCA results (Figure 3G) suggested that the species structure of each group was similar.

### 3.8. Gut Microbial Abundance Changes

#### 3.8.1. *Bacteroidaceae* Abundance

**Figure 4:**
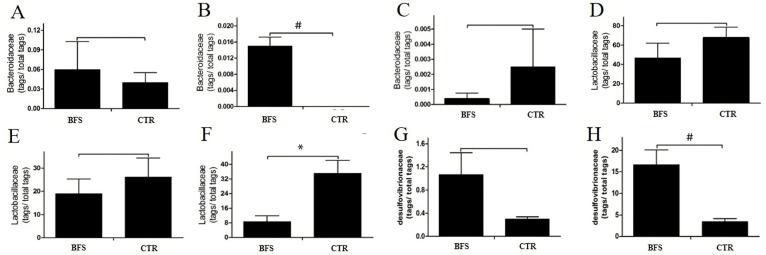
Flora abundance change versus time. (**A**–**C**): *Bacteroidaceae* tags/total tags ratio versus time. (**A**): Before supplementation. (**B**): At the 2nd week after supplementation. (**C**): At the 12th week after supplementation. (**D**–**F**): *Lactobacillus* tags/total tags ratio versus time. (**D**): Before supplementation. (**E**): At the 2nd week after supplementation. (**F**): At the 12th week after supplementation. (**G**,**H**): *Desulfovibrionaceae* tags/total tags ratio versus time. (**G**): At the 2nd week after supplementation. (**H**): At the 12th week after supplementation. * *p* < 0.05, # *p* < 0.01 compared with the CTR group.

The *Bacteroidaceae* (BAC) abundance in the intestinal tract of mice decreased gradually with age. At the family level, the BAC abundance values in the BFS and control groups were 0.06% ± 0.0096% and 0.04% ± 0.0034% (*p* > 0.05), respectively, before supplementation (Figure 4A). BAC was not detected in the feces of the control group, but BAC abundance was 0.015% ± 0.015% in the BFS group (*p* < 0.01) at the 2nd week after supplementation (Figure 4B). At the 12th week (Figure 4C), the BAC abundance values in the feces of BFS and control groups were 0.0003% ± 0.00006% and 0.0020% ± 0.00045% (*p* > 0.05), respectively.

#### 3.8.2. *Lactobacillaceae* Abundance

At the family level, the *Lactobacillaceae* (LAC) abundance values in the feces of mice in the BFS and control groups before supplementation were not significantly different (46.93% ± 34.11% vs. 67.74% ± 24.15%, *p* > 0.05) (Figure 4D). LAC abundance 4 weeks after supplementation significantly decreased in both groups compared with that before supplementation. LAC abundance decreased from 46.93% to 19.04% in the BFS group and from 67.74% to 26.17% in the control group (Figure 4E). LAC abundance in both groups continued to decline with age. Compared with that in the control group, LAC abundance in the BFS group significantly decreased (35% ± 14% vs. 9% ± 6%, *p* < 0.05) at 12 weeks after supplementation (Figure 4F).

#### 3.8.3. *Desulfovibrionaceae* Abundance

At the family level, the *Desulfovibrionaceae* (DSV) was not detected in the feces of mice in both groups before supplementation. At 4 weeks after supplementation, DSV appeared in the feces of mice, but no significant difference was observed in both groups (1.07% ± 0.84% vs. 0.30% ± 0.08%, *p* > 0.05; Figure 4G). Subsequently, the DSV abundance increased with age. At 12 weeks, the DSV abundance in the BFS group was significantly higher than that in the control group (16.67% ± 5.98% vs. 3.49% ± 1.32%, *p* < 0.01; Figure 4H).

## 4. Discussion

In this study, we first reported the gut microflora dysbacteriosis induced by BFS, which was characterized by reduced LAC and increased DSV abundance values. Glucose and lipid metabolic disorders were aggravated, and the inflammatory response was activated. This phenomenon ultimately worsened the plaque formation and atherosclerotic progression in the aorta in the mice animal model.

The abundance of *B. fragilis* is evidently influenced by various factors, including diet, physical condition, drug intake, and lifestyle habits. A diet containing high protein, fat, and carbohydrate leads to significant changes in *B. fragilis* abundance [15,16]. *B. fragilis* abundance in the gut microbiome usually has a positive correlation with obesity, inflammatory bowel disease, and colorectal cancer [17,18,19,20,21] due to the pathogenesis of enterotoxigenic *B. fragilis* (ETBF) strains that harbor *B. fragilis* toxin (BFT) genes encoding BFT [22]. ETBF strains can pathogenically disrupt the intestinal mucosa and toxin regulator system and subsequently induce energy metabolism dysfunction and intestinal/extraintestinal disorders, including intestinal infections, inflammatory bowel disease, and systemic inflammation [23]. ETBF/BFT can stimulate β-actin–T-cell factor nuclear signaling that is regulated by γ-secretase, Wnt, and NF-κB pathways through a colonic epithelial receptor, thereby inducing E-cadherin cleavage in the intestinal epithelium [24]. The proto-oncoprotein c-Myc expression, inflammation, DNA damage, and ultimately, abnormal cell proliferation are promoted [18,25,26,27]. Moreover, ETBF can enter the bloodstream due to intestinal dysbiosis and barrier dysfunction. The extreme proinflammatory cytokines induced by *B. fragilis* lipopolysaccharide (BF-LPS) leak into the blood via the breaches in the gastrointestinal tract and ultimately induce the systemic inflammation of the host by promoting the expressions of NF-κB complex, inflammatory transcription factor, and proinflammatory microRNAs mediated by the Toll-like receptor (TLR) 2, TLR4, and/or CD14 signaling pathways [28,29]. BF-LPS can increase interleukin (IL)-8 secretion, E-selectin expression, and monocyte adhesion in coronary artery endothelial cells and consequently accelerate atherosclerotic progression [30]. In our previous study, we found that *B. fragilis* abundance is markedly increased in patients with CHD and that such abundance was positively correlated with their hip circumference. The present study demonstrated that BFS increased food intake, blood glucose, excessive energy, and rapid weight gain in mice. Furthermore, LDL significantly increased, which contributed to atherosclerotic progression. Systemic inflammatory factors CD36 and F4/80 are commonly used for evaluating the markers of atherosclerotic progression [31,32,33,34]. Chronic low-grade inflammation induced by the gut microbiota is a potential driver of atherosclerotic cardiovascular disease in humans [35]. In this study, BFS dramatically increased the CD36 and F4/80 expression levels, which potentially participated in arterial endothelial cell dysfunction and subsequently promoted the atherosclerotic progression in mice. Therefore, the aggravation of aorta atherosclerosis resulted from overeating, aberrant glucose and lipidemia metabolism, and systemic inflammation, which were induced by BFS.

The gut microflora is affected by host, diet, and interaction among microflora species and undergoes the stock shock period from birth to weaning in mice. At this time, the composition and abundance of gut microflora are evidently affected by the diet and environment. With the formation of the dietary habits of mice, the gut microflora stabilizes, especially after 6–8 weeks. Abnormal interactions among different microflora species potentially influence the internal environment of the body and contribute to the pathogenesis of obesity, lipid metabolism, inflammation, and subsequent atherosclerosis. For example, daily LAC intake for 6 months significantly decreases *B. fragilis* abundance and body weight and improves the lipid metabolism in obese children, thereby elevating HDL level [36]; these phenomena suggest that increased LAC and decreased *B. fragilis* abundance levels alleviate the risk factors of atherosclerosis. In the present study, the gut microflora in mice at the time of weaning were composed of Enterobacteriaceae, LAC, *Helicobacteraceae*, and BAC. Aberrant changes in gut microflora, which were characterized by decreased LAC abundance and increased DSV abundance with age in the feces of mice, were aggravated by BFS. According to previous research, LAC supplementation can improve food digestibility, regulate lipid metabolism, reduce serum cholesterol concentration, and prevent LDL oxidation. LAC can depress gut spoilage products, inhibit spoilage bacteria growth and reproduction, and reduce endotoxin production and its damage to the body [37]. Moreover, LAC plays an antioxidative role by scavenging reactive oxygen species and free radicals and activating the antioxidant system [38,39,40,41]. LAC also releases vasodilator factors and reduces circulating blood pressure. Therefore, the increase in LAC abundance has an anti-atherosclerotic effect, and the BFS-induced decrease in LAC abundance worsens the progression of aorta atherosclerosis.

**Figure 5:**
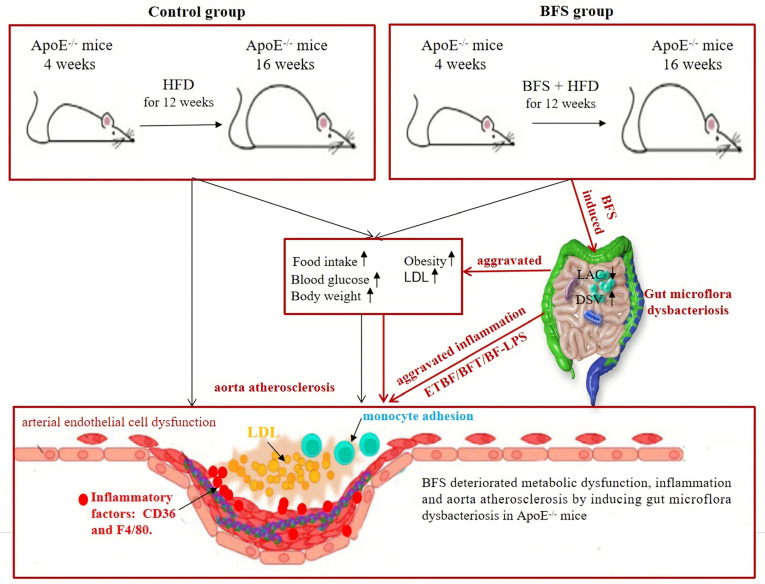
The deteriorating mechanisms of aorta atherosclerosis induced by *Bacteroides fragilis* supplementation. HFD: high-fat diet; BF: *Bacteroides fragilis*; BFS: *Bacteroides fragilis* supplementation; LAC: *lactobacillaceae*; DSV: *desulfovibrionaceae*; IL-8: interleukin-8; LDL: low-density lipoprotein cholesterol.

DSV, a kind of obligate anaerobic Gram-negative and sulfate-reducing bacteria, can reduce sulfate and produce H_2_S gas during cell growth. HFD drives hepatic inflammation by inducing the alteration of gut microbiota and metabolites in mice, especially the increase in DSV abundance; this change was consistent with the increase in the mRNA levels of CD36 and TLR4 [42,43]. The increase in DSV abundance in the gut changes the composition and structure of gut microecology and alters the physiologically steady state of gut microflora. DSV metabolites can promote the secretion of inflammatory factors, such as IL-6 and IL-8, thereby triggering a systemic inflammatory response [44,45]. This response induces the apoptosis of vascular endothelial cells, which is an important mechanism underlying atherosclerosis. DSV further aggravates atherosclerosis by affecting blood pressure, obesity, lipid metabolism, and insulin resistance [46,47]. Therefore, the increase in DSV abundance also contributes to the pathogenesis of worsening atherosclerotic progression induced by BFS. As mentioned above, the increased DSV abundance and decreased LAC abundance potentially result in the dramatic worsening of obesity, glucose, and lipid metabolic disorders. Inflammatory response is the most common risk factor that induces vascular endothelial cell dysfunction, which consequently aggravates vascular plaque formation and atherosclerotic progression (Figure 5).

Our study has several limitations. For example, we did not detect the ETBF/BFT and BF-LPS levels in the gut and plasma and inflammatory factors in plasma. However, previous studies indicated that the increase in *B. fragilis*, decrease in LAC, and elevated DSV abundance led to abnormal glucose/lipid metabolism and inflammatory response, which can also explain the relationship between the change in abundance of *B. fragilis*, LAC, and DSV and the aggravation of atherosclerosis.

## 5. Conclusions

Conclusively, we demonstrated for the first time that BFS by gavage induced gut microflora dysbacteriosis, which was characterized by decreased LAC abundance and increased DSV abundance. This condition led to the dramatic deterioration of glucose and lipid metabolic disorders and inflammation. This phenomenon aggravated vascular plaque formation and atherosclerotic progression in the mice animal model.

## Figures and Tables

**Table 1:** Full length amplification primers of 16s rDNA genome.

	Primer Sequence
520F2	AATGATACGGCGACCACCGAGATCTACACTCTTTCCCTACACGACGCTCTTCCGATCTACGATGCTAYTGGGYDTAAAGNG
520F3	AATGATACGGCGACCACCGAGATCTACACTCTTTCCCTACACGACGCTCTTCCGATCTAGACTGTCAYTGGGYDTAAAGNG
520F4	AATGATACGGCGACCACCGAGATCTACACTCTTTCCCTACACGACGCTCTTCCGATCTAGCATCGTAYTGGGYDTAAAGNG
520F5	AATGATACGGCGACCACCGAGATCTACACTCTTTCCCTACACGACGCTCTTCCGATCTATCGTAGCAYTGGGYDTAAAGNG
520F8	AATGATACGGCGACCACCGAGATCTACACTCTTTCCCTACACGACGCTCTTCCGATCTCGTAGCATAYTGGGYDTAAAGNG
520F9	AATGATACGGCGACCACCGAGATCTACACTCTTTCCCTACACGACGCTCTTCCGATCTCTACGATGAYTGGGYDTAAAGNG
520F10	AATGATACGGCGACCACCGAGATCTACACTCTTTCCCTACACGACGCTCTTCCGATCTGACAGTCTAYTGGGYDTAAAGNG
520F11	AATGATACGGCGACCACCGAGATCTACACTCTTTCCCTACACGACGCTCTTCCGATCTGCATCGTAAYTGGGYDTAAAGNG
520F12	AATGATACGGCGACCACCGAGATCTACACTCTTTCCCTACACGACGCTCTTCCGATCTGTACTGCAAYTGGGYDTAAAGNG
520F13	AATGATACGGCGACCACCGAGATCTACACTCTTTCCCTACACGACGCTCTTCCGATCTGTAGCATCAYTGGGYDTAAAGNG
520F14	AATGATACGGCGACCACCGAGATCTACACTCTTTCCCTACACGACGCTCTTCCGATCTTACGATGCAYTGGGYDTAAAGNG
520F15	AATGATACGGCGACCACCGAGATCTACACTCTTTCCCTACACGACGCTCTTCCGATCTTCACGAGTAYTGGGYDTAAAGNG
520F16	AATGATACGGCGACCACCGAGATCTACACTCTTTCCCTACACGACGCTCTTCCGATCTTCGACTAGAYTGGGYDTAAAGNG
803R3	CAAGCAGAAGACGGCATACGAGATGCCTAAGTGACTGGAGTTCAGACGTGTGCTCTTCCGATCTCTACCRGGGTATCTAATCC
803R4	CAAGCAGAAGACGGCATACGAGATTGGTCAGTGACTGGAGTTCAGACGTGTGCTCTTCCGATCTCTACCRGGGTATCTAATCC
803R5	CAAGCAGAAGACGGCATACGAGATCACTGTGTGACTGGAGTTCAGACGTGTGCTCTTCCGATCTCTACCRGGGTATCTAATCC
803R6	CAAGCAGAAGACGGCATACGAGATATTGGCGTGACTGGAGTTCAGACGTGTGCTCTTCCGATCTCTACCRGGGTATCTAATCC
803R7	CAAGCAGAAGACGGCATACGAGATGATCTGGTGACTGGAGTTCAGACGTGTGCTCTTCCGATCTCTACCRGGGTATCTAATCC
803R9	CAAGCAGAAGACGGCATACGAGATCTGATCGTGACTGGAGTTCAGACGTGTGCTCTTCCGATCTCTACCRGGGTATCTAATCC
520F2	AATGATACGGCGACCACCGAGATCTACACTCTTTCCCTACACGACGCTCTTCCGATCTACGATGCTAYTGGGYDTAAAGNG
502F7	AATGATACGGCGACCACCGAGATCTACACTCTTTCCCTACACGACGCTCTTCCGATCTCGATGCTAAYTGGGYDTAAAGNG
803R12	CAAGCAGAAGACGGCATACGAGATTACAAGGTGACTGGAGTTCAGACGTGTGCTCTTCCGATCTCTACCRGGGTATCTAATCC

**Table 2:** Primer sequences.

Gene			Primers	Length (bp)
F4/80	F	5′	GAATACAGAGACGGGGTTTA3′	198
	R	5′	CGTGTCCTTGAGTTTAGAGA3′	
Tlr2	F	5′	TTTTCACCACTGCCCGTA3′	144
	R	5′	CAGCTCGCTCACTACGTC3′	
CD36	F	5′	AAGCCAGCTAGAAAAATAG3′	184
	R	5′	AAGCCAGCTAGAAAAATAG3′	
Tlr4	F	5′	GCTTTCACCTCTGCCTTCACTAC3′	172
	R	5′	GACACTACCACAATAACCTTCCG3′	
GAPDH	F	5′	CTTTGGCATTGTGGAAGGGCTC3′	194
	R	5′	GCAGGGATGATGTTCTGGGCAG3′	

**Table 3:** Effects of *bacteroides fragilis* supplementation on body weight, heart weight and ejection fraction in mice.

Index	Bacteroidesfragilis Supplementation	Control Group	*p* Value
body weight (g)	28.36 ± 1.55	24.18 ± 1.83	0.005
heart weight (g)	0.19 ± 0.014	0.15 ± 0.027	0.016
heart weight/body weight (mg/g)	6.90 ± 0.72	6.25 ± 0.75	0.201
EF (%)	68.39 ± 3.74	66.24 ± 5.24	0.308

## Data Availability

The data and material that support the findings of this study are available from the corresponding author upon reasonable request. Anonymized data will be shared by request from any qualified investigator.
